# Detection of (1,3)-β-d-Glucan for the Diagnosis of Invasive Fungal Infection in Liver Transplant Recipients

**DOI:** 10.3390/ijms18040862

**Published:** 2017-04-19

**Authors:** Eric Levesque, Fadi Rizk, Zaid Noorah, Nawel Aït-Ammar, Catherine Cordonnier-Jourdin, Sarra El Anbassi, Christine Bonnal, Daniel Azoulay, Jean-Claude Merle, Françoise Botterel

**Affiliations:** 1 Department of Anaesthesia and Surgical Intensive Care-Liver ICU, AP-HP Henri Mondor Hospital, 51 Avenue du Marechal de Lattre de Tassigny, 94100 Créteil, France; fadi.a.risk2@gmail.com (F.R.); zaid.noorah@gmail.com (Z.N.); jean-claude.merle@aphp.fr (J.-C.M.); 2 INSERM, Unit U955, 94100 Creteil, France; 3 Mycology Unit–Microbiology department, DHU “Virus, Immunité, Cancer” VIC, AP-HP Henri Mondor Hospital, 94100 Creteil, France; nawel.ait-ammar@aphp.fr (N.A.-A.); sarra.elanbassi@gmail.com (S.E.A.); christine.bonnal@aphp.fr (C.B.); francoise.botterel@aphp.fr (F.B.); 4 EA Dynamyc Université Paris-Est Créteil (UPEC), Ecole National Vétérinaire d’Alfort (ENVA), Faculté de Médecine de Créteil, 8 rue du Général Sarrail, 94010 Créteil, France; 5 Pharmacy Unit, AP-HP Henri Mondor Hospital, 51 Avenue du Maréchal De Lattre de Tassigny, 94010 Créteil, France; catherine.cordonnier-jourdin@aphp.fr; 6 Digestive Surgery and Liver Transplant Unit, AP-HP Henri Mondor Hospital, 94100 Créteil, France; daniel.azoulay@aphp.fr

**Keywords:** (1,3)-β-d-glucan, invasive fungal infection, liver transplantation, invasive pulmonary aspergillosis, invasive candidiasis

## Abstract

Invasive fungal infections (IFI) are complications after liver transplantation involving high morbidity and mortality. (1,3)-β-d-glucan (BG) is a biomarker for IFI, but its utility remains uncertain. This study was designed to evaluate the impact of BG following their diagnosis. Between January 2013 and May 2016, 271 liver transplants were performed in our institution. Serum samples were tested for BG (Fungitell^®^, Associates Cape Code Inc., Falmouth, MA, USA) at least weekly between liver transplantation and the discharge of patients. Nineteen patients (7%) were diagnosed with IFI, including 13 cases of invasive candidiasis (IC), eight cases of invasive pulmonary aspergillosis, and one case of septic arthritis due to *Scedosporium apiospernum*. Using a single BG sample for the primary analysis of IFI, 95% (21/22) of the subjects had positive BG (>80 pg/mL) at the time of IFI diagnosis. The area under the ROC curves to predict IFI was 0.78 (95% CI: 0.73–0.83). The sensitivity, specificity, positive predictive value (PPV) and negative predictive value (NPV) of BG for IFI were 75% (95% CI: 65–83), 65% (62–68), 17% (13–21), and 96% (94–97), respectively. Based on their high NPV, the BG test appears to constitute a good biomarker to rule out a diagnosis of IFI.

## 1. Introduction

Patients undergoing liver transplantation (LT) are specifically at risk of developing an invasive fungal infection (IFI) (5–10% of cases) during the early post-operative period (≤2 months) [1,2]. The most common invasive fungal pathogen is *Candida* sp., followed by *Aspergillus* sp. [3,4,5]. The early identification and treatment of an IFI are challenging and a delayed or missed diagnosis is associated with higher morbidity and mortality [1,5,6]. The lack of sensitivity and specificity of the diagnostic tests available at present is the principal contributor to a delay in diagnosis. For this reason, non-culture-based biomarkers to detect circulating fungal cell wall constituents have been developed during the past twenty years. One such diagnostic assay is (1,3)-β-d-glucan (BG). This fungal wall component is detected in many IFI, including *candidiasis*, *aspergillosis*, and *Pneumocystis jirovecii* pneumonia (*PjP*), but cannot be used for *Cryptococcus* and *Mucorales* infections. Several studies, systematic reviews, and meta-analyses have focused on the accuracy of the serum BG assay for the diagnosis of IFI [7,8,9,10]. Some of them showed that the detection of BG displayed good sensitivity and specificity for IFI in the general population, even if data are variable according to the population studied [11].

In the context of LT, the utility of this test has been poorly documented. During a prospective study to evaluate a pre-emptive strategy based on BG levels in LT, Akamatsu et al. observed a high sensitivity associated with a high negative predictive value (NPV) [12]. However, recently, Singh et al. found that given the high rates of false positive results, BG was of limited utility in diagnosing IFI after LT [13]. These authors observed that the baseline marker was positive in approximately 90% of cases, the results were centre-dependent, and 50% of patients continued to have positive results up to four weeks later [13]. Nevertheless, the performance of BG was dependent on the cut-off value applied. In a previous preliminary study, we found that BG could constitute a promising tool to rule out invasive candidiasis in high-risk liver transplant recipients, the optimum diagnostic performance being achieved with a value of 146 pg/mL [14]. However, this study was limited by the low incidence of IFI cases and a small sample size.

On the occasion of the retrospective analysis of our IFI, we, therefore, prospectively evaluated BG levels in a large monocentric cohort of liver transplant recipients and their performance with respect to the detection of IFI.

## 2. Results

### 2.1. Clinical Characteristics

Between January 2013 and May 2016, 277 consecutive patients underwent LT, six of which were excluded from the analysis in this study because of urgent re-transplantation for hepatic artery thrombosis or primary non-function. Two hundred seventy-one transplants were included in our study and performed in 262 patients (259 liver transplantations, 12 liver-kidney transplantations). Nine patients required re-transplantation at least three months after the first LT. The characteristics and risk factors for IFI at the time of transplantation are shown in Table 1. The median age of the study population was 52.7 ± 12.7 years, and 202 of the patients (74.5%) were male. The mean model for end-stage liver disease (MELD) score of patients was 19.1 ± 11.4.

### 2.2. Prophylaxis Treatment

One hundred and fifty-seven liver recipients (58%) received antifungal prophylaxis with caspofungin (*n* = 132; 49%), micafungin (*n* = 6; 2.2%), fluconazole (*n* = 18; 6.6%), or amphotericin B (*n* = 1; 0.4%), in a patient with a history of pulmonary mucormycosis. The mean duration of prophylaxis was 24.3 ± 18.2 days. Among the classical risk factors for IFI occurrence post-LT, the recipients with a MELD score of 30 at the time of LT and early re-intervention were the most common (*n* = 51, 19%), followed by renal replacement therapy (*n* = 44, 16%) and choledocojejunostomy anastomosis (*n* = 38, 14%).

Seventeen patients (10.8%) received prophylactic treatment for longer than 28 days because of the persistence of at least one risk factor (renal replacement therapy *n* = 11; re-intervention *n* = 4 or chronic graft dysfunction *n* = 3). The total cost of this prophylaxis was €1,614,796, or around €10,200 per liver transplant.

### 2.3. Invasive Fungal Infections

Out of a total of 271 liver recipients, 19 (7%) developed one or more IFI during their hospital stay. *Candida* and *aspergillus* infections occurred in 4.05% and 3.3% of patients, respectively, with one patient developing both invasive candidiasis (IC) and invasive pulmonary aspergillosis (IPA) during the same stay.

A total of thirteen cases of IC were found in eleven recipients (patients 1–11). Twelve infections were proven and one was probable (Table 2). Among these infections, eight were proven to be intra-abdominal candidiasis involving *C. glabrata* (*n* = 2), *C. albicans* (*n* = 2), *C. krusei* (*n* = 1) or a combination of two *Candida (C. albicans/C. glabrata* and *C. albicans/C. krusei)* (*n* = 2) or three *Candida (C. glabrata*, *C. albicans*, *C. tropicalis)* (*n* = 1), in the context of gastrointestinal perforation or biliary abscess. Four patients had positive blood cultures with *C. albicans* (*n* = 2), *C. parapsilosis* (*n* = 1) or *C. orthopsilosis* (*n* = 1). At the time of IC (*n* = 13), nine patients received prophylactic therapy (with caspofungin or fluconazole), and in all cases were sensitive to this treatment.

Seven liver recipients (patients 12–18) experienced probable IPA, according to the European Organization for Research and Treatment of Cancer/Mycoses Study Group (EORTC/MSG) criteria (Table 3). In addition, one patient developed IPA following IC (patient 2). All recipients, at the time of the diagnosis of IA, were receiving antifungal prophylaxis with caspofungin. After the diagnosis, all patients were treated with voriconazole (5 mg/kg twice daily, adjusted to blood concentrations) for a period of 23 ± 8 days. Finally, one patient (patient 19) developed proven septic arthritis of the hip caused by *Scedosporium* spp.

### 2.4. IFI and Survival

**Figure 1:**
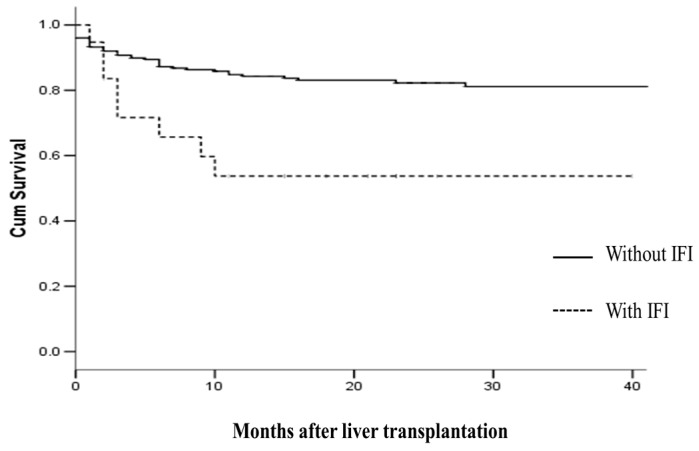
Patient survival following liver transplantation as a function of invasive fungal infection status, *p* = 0.003.

The median ICU and hospital stays after LT were 7.9 ± 9.8 and 33.8 ± 28.2 days, respectively. The median follow-up period was 20 months, and no patient was lost to follow-up. Notwithstanding the difficulty of assessing the primary cause of death in these recipients, which was sepsis/multi-organ failure in 73% of cases, no deaths appeared directly to be due to IFI. Patient survival rates at six months and one year after LT were 87% and 83.4%, and graft survival rates were 85% and 81.6%, respectively. Recipients with IFIs had significantly lower patient survival rates at six months and one year when compared to recipients with no IFI (89.3% vs. 68.9%; *p* = 0.007 at six months, and 85.3% vs. 57.9%; *p* = 0.002 at one year, respectively). Finally, Kaplan Meier curves were fitted for the two groups of patients according to the IFI experienced, and compared using the log-rank test (Figure 1).

### 2.5. BG Analysis

For the BG test, 1019 samples were obtained from the 236 recipients. Thus, the average number of samples obtained from each patient was 4.31 (range: 1 to 18), or about one per week. In 35 recipients without IFI risk factors and a short ICU stay, no sample was collected during their stay. None of these recipients developed any fungal infections. Using the cut-off of 80 pg/mL, the BG positivity was 48% (113/236 patients) at baseline, 35% (83/236 patients) at week 1 and 30% (56/189 patients) at week 2 (Table 4). In patients without IFI, 34% (318/924 samples) were over 80 pg/mL. Factors associated with BG positivity at the time of sampling were: renal replacement therapy (*n* = 56 samples from 22 patients), surgery (LT or repeat surgery <5 days) (*n* = 196 samples from 121 patients), the administration of piperacillin-tazobactam (*n* = 66 samples from 52 patients) and, in 25 positive samples, neither IFI nor factors associated with BG positivity was found. During the follow-up of patients without IFI, the BG concentration is significantly different between patients receiving echinocandin prophylaxis (*n* = 124 patients with 375 samples) and patients without echinocandin prophylaxis (*n* = 131 patients with 304 samples) (109 ± 116 vs. 89 ± 95 pg/mL, *p* < 0.001).

### 2.6. IFI and BG

When applying the cut-off value of 80 pg/mL, and using a single BG sample for the primary analysis of IFI, 95% (21/22) of the subjects with IFI had positive BG at the time of IFI diagnosis (between −7 and +7 days of onset of clinical symptoms of IFI) (Table 4). The mean BG concentration during the 28-day diagnostic window for recipients with proven or probable IFI was significantly higher than the mean value seen in those without IFI (244 ± 173 vs. 99 ± 106 pg/mL, respectively; *p* < 0.001).

**Figure 2:**
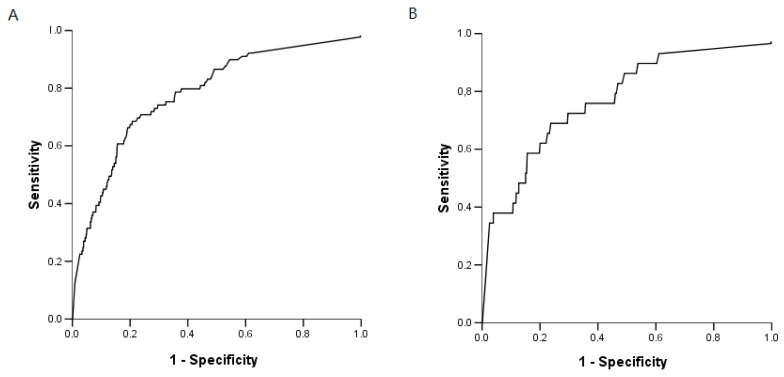

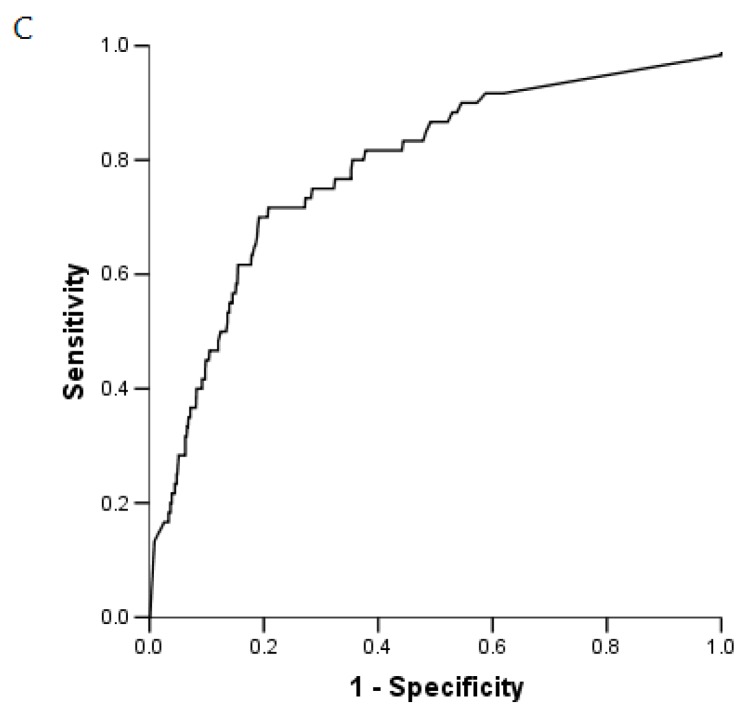
Receiver operating characteristic (ROC) curves used to evaluate the ability of BG to diagnose (**A**) an invasive fungal infection (IFI): AUROC (Area under ROC) value of 0.78 (95% confidence interval (CI): 0.73–0.83); (**B**) Invasive pulmonary aspergillosis (IPA): AUROC value 0.77 (95% CI: 0.67–0.86); and (**C**) invasive candidiasis (IC): AUROC value 0.78 (95% CI: 0.72–0.84).

Receiver operating characteristic (ROC) curves were used to evaluate the potential for BG to diagnose IFI (Figure 2). The area under the ROC curves was 0.78 (95% CI: 0.73–0.83). The sensitivity, specificity, positive predictive value (PPV) and negative predictive value (NPV) of BG for IFI, overall, were 75% (95% CI: 65–83), 65% (95% CI: 62–68), 17% (95% CI: 13–21), and 96% (95% CI: 94–97), respectively. If IC and IPA were considered separately, the same results in terms of AUROC (area under receiver operating characteristic) were observed: the AUROC value was 0.77 (5% CI: 0.67–0.86) for the diagnosis of IPA and 0.78 (95% CI: 0.72–0.84) for the diagnosis of IC (Figure 2).

In recipients with IFI, the median time elapsing between the first clinical signs of IFI and a BG value ≥ 80 pg/mL was −2 days (range: −7 to 14 days). The peak BG value was reached after a median of four days (range: −7 to 28 days) after the diagnosis of IFI. After antifungal treatment, the negativity of BG (below 80 pg/mL) was obtained in 66% (14/21 patients) ranged from six to 21 days after the BG peak (median 13 days). In addition, the BG decreases with the antifungal treatment in 90% of the subjects (19/21).

## 3. Discussion

In this report, we investigated the incidence of IFI in the era of antifungal prophylaxis and assessed the performance of BG in diagnosing IFI among liver transplant recipients. Out of a total of 271 recipients, 19 (7%) (who experienced twenty-one episodes) developed proven or probable IFI during their ICU stay.

The incidence of IFI in our patients receiving targeted prophylactic antifungal therapy was 7%, but this reached 12% among liver recipients with high risk of IFI. Our result was similar to the incidence reported elsewhere, with rates reaching 17.7% [1,4,15,16,17,18,19,20,21]. In the era of MELD and targeted prophylactic antifungal therapy, our study was able to offer three interesting pieces of information: (i) all patients who developed IFI within two months of LT had one or more risk factors and were receiving antifungal therapy after LT; (ii) the time to onset of IFI in the ICU after LT ranged from five days to 252 days. In eight cases, IFI occurred before the tenth post-operative day due to IC (*n* = 5, associated with surgical complications) or IPA (*n* = 3). These IA were probably present before LT but had not been diagnosed. This raises the question regarding the need to determine the presence of IA in end-stage liver patients with a high MELD score or organ failure, because this may reflect a greater severity of their liver disease [17]; and (iii) our findings confirm that IFI represent a significant burden in terms of mortality and morbidity, and highlight the importance of their early detection.

The BG assay has been shown to be useful for the diagnosis of IFI in haematological patients [22,23,24]. In LT, only a few studies have to date evaluated the utility of BG. During a preliminary study, we investigated the impact of the systematic monitoring of BG levels in liver transplant recipients. We observed that serum BG levels achieved good diagnostic accuracy for the diagnosis of IC [14]. The principal limitation of that preliminary study was the low incidence of IFI cases (six episodes of invasive candidiasis out of a total of 52 patients). In this new study, concerning a large patient sample and 21 episodes of IFI, our findings agree with our previous results and with those obtained by Akamatsu et al. [12,14]. In all three studies, the AUROC curves for the detection of IFI using BG ranged from 0.72 to 0.79. By contrast, Singh et al. found that the BG test was ineffective in predicting IFI, with an AUROC curve of 0.56 [13]. This was due to the numerous false positive tests observed in their study. Indeed, the baseline marker (after surgery) was positive at about 90% in the Singh study, but declined over time, with approximately 50% of the patients still having positive tests up to four weeks later [13]. A number of factors can cause the false positivity of this marker in the clinical setting: patients who are receiving renal replacement therapy, albumin or immunoglobulin infusions, β-lactam inhibitors, antibiotics, surgical gauze, etc. [25,26,27,28,29]. In our study, the baseline marker (just after LT) was positive in only half of recipients and declined over time. Moreover, in 92% of BG positivity, at least one factor associated with BG positivity was found (renal replacement therapy, after surgery, the administration of piperacillin-tazobactam). Finally, we observe that an echinocandin treatment can increase the BG value, by release of BG, but in order to reduce this high proportion of false positives, we have proposed confirming a positive result by testing rapidly (or quickly) a second sample or to increase the cut-off value rather than 80 pg/mL [14]. The most important finding of our study is that the IFI diagnosis may be excluded when the BG value is less than 80 pg/mL. In this case, as shown by Singh et al. [13], this test has a sensitivity close to 100% (BG values were higher than 80 pg/mL in 95% of IFI cases (20/21 episodes).

We are aware of the limitations of this study. Nearly half of the recipients in our study received antifungal prophylaxis because of their risk factors, but without evident signs of an IFI. They may have been affected by a latent infection that was treated with the antifungal therapy. The BG had to also be affected by echinocandin treatment, probably by the release of BG. In addition, the prophylaxis in high-risk patients may have directly affected the estimations of BG efficacy in this population. For these reasons, our results can only be extrapolated to patients who were being treated initially with echinocandin (90% of antifungal prophylaxis). Furthermore, our study was not designed to assess BG as a predictor of survival.

## 4. Patients and Methods

This study was carried out in liver transplant patients at Henri Mondor Hospital in the suburbs of Paris, France. Between January 2013 and May 2016, all patients admitted consecutively to our Intensive Care Unit (ICU) following LT were enrolled in the study. Demographic and clinical characteristics, investigations for mycological and bacterial infections, and antimicrobial therapies were all recorded prospectively. The patients were studied during their hospitalization in and after the ICU. Our Institutional Review Board approved the study (2012-12) and the database was declared to the French Data Protection Authority (Commission Nationale Informatique et Liberté) (No. 1699340).

### 4.1. Clinical and Biological Management

Following LT, the recipients were hospitalized in our liver ICU. All patients received similar post-operative intensive care with a standard triple immunosuppressive regimen that included corticosteroids, mycophenolate mofetil and FK506 (tacrolimus) or cyclosporin, with basiliximab (day 1 and day 4) in the event of a rising, or initially high, serum creatinine level. All patients received post-operative antibiotic therapy for 48 h (piperacillin) and trimethoprim-sulfamethoxazol (TMP/SMX) as prophylaxis against PjP in high-risk patients (fulminant hepatitis, re-transplantation). To prevent cytomegalovirus (CMV) disease, pre-emptive therapy was instituted. The patients were monitored for evidence of CMV replication, and antiviral therapy (valganciclovir or ganciclovir) was administered pre-emptively, if necessary, to prevent progression to symptomatic clinical disease. Graft and recipient outcomes were recorded prospectively for all transplanted patients. The clinical course of recipients was followed for a minimum of three months after LT.

### 4.2. Antifungal Prophylaxis

Identification of patients at increased risk of fungal infection (re-transplantation, renal failure (creatinine clearance <50 mL/min or requiring replacement therapy), fulminant hepatic failure, primary non-function, patients with thymoglobulin as immunosuppressive agents, complicated surgery or reoperation, recipient MELD scores before LT > 30, more than 40 pre-operatory transfusion blood products, bilio-digestive anastomosis and multifocal colonization (≥2 localisations) by Candida spp., and the yeast preservation fluid contamination) is the key of prevention [14,30]. Patients affected by any of these factors received caspofungin at a dose of 70 mg on the first day, and then 50 mg per day (or 70 mg per day if the recipient weighed >80 kg), or micafungin at a dose of 100 mg per day for 28 days. The choice between capsofungin and mcafungin was left to the discretion of the physician. The few patients who were only considered to be at risk of Candida infection, but not Aspergillus (patients with choledocho-jejunostomy or colonized at the time of liver transplantation by *Candida* spp.), received fluconazole 400 mg per day for three weeks in the absence of *Candida glabrata*, *Candida krusei*, or the prior use of azoles.

### 4.3. Colonisation

All patients were subject to weekly monitoring for *Candida* colonization and were screened for *Candida* infections in blood cultures and at other sterile sites, depending on their clinical signs. As part of this routine surveillance of *Candida* colonization, swabs were systematically taken from five superficial sites (mouth, nose, axillary surface, inguinal fold, and anus) on the day of admission to undergo LT, and then once a week thereafter until discharge from the ICU or death. Colonisation is defined as the presence of *Candida* spp. isolates in a swab from at least one surveillance site.

### 4.4. Galactomannan

Serum from all patients at risk of invasive pulmonary aspergillosis (IPA) was screened twice weekly for galactomannan (Platelia *Aspergillus*, Biorad, Marne la Coquette, France) during the patient's ICU stay. The threshold was 0.5, according to the revised European Organization for Research and Treatment of Cancer/Mycoses Study Group (EORTC/MSG) criteria [31]. If the patients had a broncho-alveolar lavage, a galactomannan assay was performed systematically at the same time as the culture.

### 4.5. (1,3)-β-d-Glucan (BG) Assay

In addition to clinical and laboratory evaluations to determine the presence of any infection, serum was collected weekly during the patient’s ICU (each monday) and according to the discretion of the physician during the hospital stay and tested for BG using the Fungitell kit (Fungitell^®^, Associates Cape Code Inc., Falmouth, MA, USA). These samples were stored at −20 °C until batch testing was performed. It was necessary for serum samples to be collected from patients with proven or probable IFI during the seven days before and 21 days after the diagnosis (thus providing a 28-day diagnostic window).

### 4.6. Definition

The diagnostic criteria used for IPA were those of the EORTC/MSG [31]. All patients were reviewed by a committee composed of four members (two intensive care clinicians and two mycologists), who classified aspergillosis as probable aspergillosis or colonization.

Invasive candidiasis (IC) included candidemia and deep-seated candidiasis, defined as the recovery of *Candida* species from blood and/or a sterile site, respectively. *Candida* infections can manifest themselves as peritonitis, candidemia, surgical anastomosis infection, urinary tract infection, or wound infection. IFI were, thus, classified according to the EORTC/MSG criteria [31].

Only patients with proven or probable IFI were evaluated as cases for the present study and the PjP were separated from the IFI.

Controls were defined as liver transplant recipients with no clinical or microbiological evidence of IC (patients only colonized by *Candida* were also considered as controls) and those free of invasive pulmonary aspergillosis.

### 4.7. Statistical Analysis

Continuous data were presented as medians and ranges. Fisher’s exact test (categorical variables) and the Mann-Whitney test (continuous variables) were used to compare demographic data based on the patient’s IC status. Kaplan-Meier curves were generated, and a log-rank test was utilized to compare patients with and without IFI. The following parameters for diagnostic performance, and their 95% confidence intervals, were calculated: sensitivity, specificity, positive predictive value (PPV), and negative predictive value (NPV). To identify the ability of BG to diagnose invasive candidiasis, receiver operating characteristic (ROC) curves were constructed. All statistical analyses were performed using SPSS (version 18; SPPS, Inc, IBM, Chicago, IL, USA). The areas under the curves (AUCs) reflected the discriminative ability of BG. A *p* value lower than 0.05 was considered to be statistically significant.

## 5. Conclusions

In a large sample of patients, the present study was able to confirm that the detection of BG in serum after LT may be a tool which can rule out IC in high-risk recipients based on its high NPV.

## Figures and Tables

**Table 1:** Characteristics of 271 liver transplants and risk factors for invasive fungal infection (IFI) at the time of transplant. Data are presented as the mean ± SD or *n* (%). HBV: viral hepatitis B; HCV: viral hepatitis C; MELD: model for end-stage liver disease; LT: liver transplantation.

Variable	Liver Transplants *n* = 271
Age (years)	52.7 ± 12.7
Gender M/F	202/69
Indication for liver transplantation	
*Hepatocellular carcinoma*	85 (31)
*Cirrhosis*	126 (46)
*Fulminant hepatic failure*	17 (7)
*Other*	43 (16)
Alcoholic liver disease	130 (48)
Viral hepatitis (HCV and HBV)	54 (20)
Other	87 (32)
MELD score	19.1 ± 11.4
MELD < 20	164 (60)
MELD 20–30	47 (18)
MELD > 30	60 (22)
Antifungal prophylaxis *n* (%)	157 (58)
Caspofungin, *n* (%)	132 (84.1)
Micafungin, *n* (%)	6 (3.8)
Fluconazole, *n* (%)	18 (11.5)
Ampho B, *n* (%)	1 (0.6)
duration of treatment (days)	24.3 ± 18.5
Risk factors for IFI	
MELD > 30	60 (22)
Fulminant hepatic failure	17 (6.3)
Re-transplantation	20 (7.5)
Graft type	
*Whole cadaveric liver transplant*	246 (91)
*Partial liver transplant (split)*	22 (8)
*Domino*	3 (1)
Multi-organ transplantation (kidney/liver)	14 (5)
>40 transfusions of blood products	8 (3)
Renal Replacement therapy	44 (16)
Early re-intervention after LT	51 (19)
Choledocojejunostomy anastomosis (Roux-en-Y)	38 (14)

**Table 2:** Characteristics of patients with invasive candidiasis. IC: invasive candidiasis, LT: liver transplantation, BG: (1,3)-β-d-glucan assay, CI: colonization index.

Patient Number	Delay between LT and IC (days)	Localisation IC	Culture	Classification of IC	Values BG (pg/mL)	CI	Prophylactic Treatment (Delay before IC)	Curative Treatment	Outcome (after LT)
1	46	Intra-abdominal	*C. glabrata*	Proven	201	0.5	Caspofungin (45)	Voriconazole	Died 301 days
	252	Candidemia	*C. parapsilosis*	Proven	501	0.8	Caspofungin (25)	Fluconazole	
2	65	Intra-abdominal	*C. glabrata*	Proven	501	0.8	Caspofungin (10)	Voriconazole	Died 81 days
3	26	Intra-abdominal candidiasis	*C. glabrata*, *C. albicans*	Proven	366	0.8	-	Caspofungin	Died 91 days
4	7	Intra-abdominal	*C. albicans*	Proven	360	0.8	Caspofungin (7)	Fluconazole	Died 47 days
5	8	Candidemia	*C. albicans*	Proven	501	0.6	Fluconazole (8)	Fluconazole	Alive 692 days
6	7	Intra-abdominal/biliary abscess	*C. albicans*	Proven	501	0.8	Caspofungin (7)	Fluconazole	Alive 677 days
7	6	Candidemia	*C. albicans*	Proven	170	0.6	Caspofungin (6)	Fluconazole	Alive 438 days
8	22	Intra-abdominal	*C. krusei*	Proven	320	0.2	Caspofungin (22)	Caspofungin	Alive 317 days
9	62	Candidemia	*C. orthopsilosis*	Proven	173	0.5	-	Liposomal Amphotericin B	Alive 177 days
10	10	Intra-abdominal	*C. krusei*, *C. albicans*	Proven	209	0.6	Caspofungin (10)	Liposomal Amphotericin B	Alive 49 days
11	12	Intra-abdominal and candidemia	*C. glabrata*	Proven	246	0.6	Caspofungin (10)	Caspofungin	Died 280 days
	86	abscess and biliary infection	*C. glabrata*, *C. albicans*, *C. tropicalis*	Probable	501	0.5	-	Caspofungin	

**Table 3:** Characteristics of patients with invasive pulmonary aspergillosis (IPA). LT: liver transplantation, BG: (1,3)-β-d-glucan assay; GM: galactomannan, BAL: bronchoalveolar lavage, VHB: viral hepatitis B; VHC: viral hepatitis C; NA: Not available.

Patient Number	Time to Onset after LT (days)	Underlying Diseases	IPA Classification	BAL Direct Examination	Culture	GM in Serum (Index)	GM in BAL (Index)	BG Values (pg/mL)	Prophylactic Treatment (Delay)	Curative Treatment	Outcome (after LT)
12	5	Cryptogenetic cirrhosis	Probable	NA	*A. fumigatus*	+ (0.602)	NA	207	Caspofungin (5)	Voriconazole	Alive at 1197 days
13	44	Acute liver failure (autoimmune hepatitis)	Probable	-	*A. fumigatus*	− (0.125)	+ (1.25)	501	Caspofungin (35)	Voriconazole	Alive at 1197 days
14	45	HCC/VHB cirrhosis	Probable	-	*A. fumigatus*	+ (>6)	+ (5.58)	198	Caspofungin (9)	Voriconazole	Died 61 days
15	7	Acute liver failure (toxic hepatitis)	Probable	NA	*A. fumigatus*	+ (1.39)	+ (1.85)	219	Caspofungin (7)	Voriconazole	Alive at 599 days
16	5	Acute liver failure Reactivation of VHB infection	Probable	-	*A. fumigatus*	− (0.06)	− (0.13)	501	Caspofungin (5)	Voriconazole	Alive at 542 days
17	32	Re-LT Chronic graft rejection	Probable	-	*A. fumigatus*	+ (2.361)	NA	42	Caspofungin (31)	Voriconazole	Alive at 41 days after LT
18	25	VHB/VHC cirrhosis	Probable	-	*A. fumigatus*	− (0.157)	+ (1.6)	170	Caspofungin (19)	Voriconazole	Died 26 days
2	55	Acute liver failure (toxic hepatitis)	Probable	-	*A. fumigatus*	+ (5.30)	+ (5.68)	423	Caspofungin (35)	Voriconazole	Died 81 days
19	22	Acute liver failure Reactivation of VHB infection	Proven Septic arthritis of the hip		*Scedosporium* sp.	+ (2.83)	NA	501	Micafungin (22)	Surgery Caspofungin + voriconazole	Alive at 599 days

**Table 4:** Kinetics of (1,3)-β-d-glucan (BG) in patients with IFI. Day 0 is defined as the beginning of clinical symptoms of IFI. IFI: Invasive fungal infection; IC: invasive candidiasis, IPA: invasive pulmonary aspergillosis, NA: not available.

IFI	Patient Number	Day 7	Day 0	Day 7	Day 14	Day 21	Day 28
IC	1	201	147	NA	NA	41	44
	1	501	356	393	501	501	446
	2	234	423	501	501	501	NA
	3	NA	36	366	NA	50	77
	4	360	464	501	NA	31	NA
	5	NA	501	269	269	191	221
	6	324	348	14	501	323	192
	7	106	153	170	110	90	51
	8	232	NA	320	311	NA	NA
	9	31	151	173	76	140	NA
	10	31	209	148	NA	NA	NA
	11	170	31	58	180	NA	246
	11	187	501	NA	NA	293	170
IPA	12	143	207	53	55	NA	NA
	13	501	NA	174	NA	NA	NA
	14	198	169	NA	NA	NA	30
	15	NA	219	128	50	NA	NA
	16	501	501	501	NA	501	501
	17	31	33	42	NA	NA	NA
	18	75	170	NA	NA	NA	NA
	2	423	131	NA	NA	NA	99
*Scedosporium* sp.	19	423	501	501	501	NA	NA

## Abbreviations

AUROC	Area Under Receiver Operating Characteristic
BG	(1,3)-β-d-glucan
CMV	Cytomegalovirus
EORTC/MSG	European Organization for the Research and Treatment of Cancer/Mycoses Study Group
IC	Invasive Candidiasis
ICU	Intensive Care Unit
IFI	Invasive Fungal Infection
IPA	Invasive Pulmonary Aspergillosis
LT	Liver Transplantation
MELD Score	Model for End-Stage Liver Disease
NPV	Negative Predictive Value
*Pj*P	*Pneumocystis jirovecii* pneumonia
PPV	Positive Predictive Value
ROC	Receiver Operating Characteristic
